# IgM-and IgG-cleaving enzyme facilitates AAV gene transfer in AAV-seropositive macaques

**DOI:** 10.3389/fimmu.2026.1966044

**Published:** 2026-10-20

**Authors:** Helen L. Wu, Zachary C. Elmore, Mark A. Ochoa, Allyson J. McCullen, Mischa K. Brown, Hannah K. Fisher, Sofia K. Potter, Mary Grace E. Mott, Joshua A. Hull, Sebastian P. Fuchs, Ava Dickinson, Lauren Bailey, Kimberly Armantrout, Christine Shriver-Munsch, Aaron Barber-Axthelm, Scott Hansen, Ronald C. Desrosiers, Aravind Asokan, Jonah B. Sacha

**Affiliations:** 1 Department of Pathobiology & Immunology, Oregon National Primate Research Center, Oregon Health & Science University, Beaverton, OR, United States; 2 Department of Surgery and Department of Molecular Genetics & Microbiology, Duke University School of Medicine, Durham, NC, United States; 3 Department of Pathology, University of Miami Miller School of Medicine, Miami, FL, United States

**Keywords:** AAV gene therapy, antibodies, cynomolgus macaque, proteases, rhesus macaque, SHIV

## Abstract

AAV gene therapy is a promising approach for long-term delivery of antiviral proteins that can control HIV replication, but pre-existing circulating AAV-specific antibodies can preclude successful AAV transduction and pose a challenge for AAV vector re-administration in cases where transgene protein expression falls below effective levels. IceMG is a novel, engineered, dual-function immunoglobulin (Ig)-cleaving enzyme that cleaves human and non-human primate IgM and IgG. Here, we sought to evaluate the ability of IceMG to transiently reduce AAV-specific humoral immunity and facilitate successful AAV transduction and gene transfer in AAV-seropositive, SHIV-infected rhesus and cynomolgus macaques. Intravenous administration of IceMG to macaques resulted in cleavage and transient depletion of circulating IgG and IgM, in contrast to dosing with IdeS, an IgG-specific immunoglobulin-cleaving enzyme, which resulted in cleavage and transient depletion of IgG but not IgM. Dosing of either IceMG or IdeS temporarily decreased SHIV Env-binding IgG plasma concentrations, which were associated with concomitant increases in SHIV plasma viral loads, underscoring the role of Env-specific humoral immunity in SHIV control. Critically, IceMG or IdeS dosing also transiently decreased AAV9-specific neutralizing antibody titers in AAV-seropositive macaques that had previously received AAV9 vectors. Re-dosing macaques with IceMG or IdeS months later reduced circulating intact IgG, SHIV Env-binding IgG titers, and AAV-specific neutralizing antibody titers with similar efficacy as the initial dose. Finally, IceMG administration to macaques prior to AAV9 vector boosting enabled a second round of successful AAV transduction and transgene expression, in contrast to macaques that did not receive IceMG where AAV vector boosting failed to produce detectable transgene. However, AAV vector boosting also led to rebound of AAV-specific neutralizing antibody titers in all macaques, and in some cases, increases in anti-drug antibodies (ADA) targeting the AAV vector transgene protein and subsequent loss of transgene protein from circulation. Overall, these results substantiate the use of Ig-cleaving enzymes as tools to overcome pre-existing AAV humoral immunity, but highlight other challenges that follow AAV vector re-administration, such as resurgence of AAV humoral immunity and AAV transgene-directed ADA.

## 1 Introduction

Adeno-associated virus (AAV)-mediated gene therapy is an effective method for long-term delivery and expression of transgenes to human hosts and has been approved for treatment of diseases such as retinal dystrophy, muscular dystrophy, hemophilia, and neurological disorders (1). AAV gene therapy is also under active investigation for prevention and treatment of HIV through delivery of transgenes encoding antiviral proteins such as virus-specific broadly neutralizing antibodies and other proteins that block viral access to cell entry receptors (2–4). We recently demonstrated that a single dose of recombinant AAV9 encoding Leronlimab, an antibody that blocks viral entry coreceptor CCR5, can result in long-term CCR5 blockade of virus-susceptible CD4+ T cells and control of SHIV replication in chronically infected rhesus and cynomolgus macaques (5).

However, pre-existing immunity to AAV poses a challenge for AAV gene therapy in the clinic as 30-80% of the population possess circulating antibodies against prevalent AAV serotypes from natural exposure (6–8). Upon administration of AAV gene therapy, pre-existing AAV-specific neutralizing antibodies (NAbs) can bind to AAV capsids and impede *in vivo* transduction, thus preventing transgene delivery (9–13). In addition, complement activation triggered by AAV capsid-specific IgM and IgG antibodies after AAV vector administration has been linked to severe inflammatory responses and toxicities (14–16). Further, re-administration or boosting with AAV vectors is challenging as the initial dose engenders AAV capsid-specific humoral immune responses in AAV-seronegative subjects and increases titers of pre-existing AAV-specific antibodies in AAV-seropositive subjects, and resulting AAV NAb titers are sustained over years (11, 12, 17–20). Thus, AAV-specific immunity must be overcome to achieve a second round of transduction and gene transfer, which may be required in cases of waning or insufficient levels of transgene protein from the initial AAV vector dose.

To overcome these challenges, recent studies have investigated using bacteria-derived immunoglobulin (Ig)-degrading/cleaving enzymes to reduce AAV-specific humoral immunity *in vivo*. Two such enzymes, IdeS and IdeZ, specifically cleave human and macaque IgG, and when administered to AAV-seropositive macaques, transiently reduce circulating AAV-specific antibodies, and enable AAV transduction (21, 22). IceMG is a novel, engineered immunoglobulin-cleaving enzyme consisting of a fusion of the proteolytic domains of IgG-cleaving enzyme IdeZ and IgM-cleaving enzyme IceM, resulting in a dual-function enzyme that cleaves both IgG and IgM (23). In a pilot study in AAV-seropositive cynomolgus macaques, IceMG simultaneously reduced the ability of antisera to activate complement and to neutralize AAV (23). Here, we sought to evaluate IceMG as a tool to reduce AAV-specific humoral immunity and facilitate AAV-mediated gene transfer in AAV-seropositive, SHIV-infected macaques.

## 2 Materials and methods

### 2.1 Research animals and animal procedures

Rhesus macaques (*Macaca* mulatta) and Mauritian-origin cynomolgus macaques (*Macaca fascicularis)* were housed at the ONPRC and utilized for studies under the approval of the Oregon Health and Science University (OHSU) West Campus Institutional Animal Care and Use Committee (IACUC) under protocols #IP00000063, #IP00003328, and #IP00004183. All rhesus macaques were negative for Mamu-A*01, B*08, B*17 alleles with the exception of NHP-4 (Mamu-A*01+, Mamu-B*08+) and NHP-7 (Mamu-B*17+). All 14 macaques were chronically infected with SHIV (see below), but did not show any clinical signs of immunosuppression, and 13 exhibited normal blood T cell counts prior to the study (T cells count in one macaque, NHP-7, not measured). All macaques in this study were managed according to the ONPRC animal care program, which is fully accredited by AAALAC International and is based on the laws, regulations, and guidelines set forth by the United States Department of Agriculture (e.g., the Animal Welfare Act and Animal Welfare Regulations, the Guide for the Care and Use of Laboratory Animals, 8^th^ edition (Institute for Laboratory Animal Research), and the Public Health Service Policy on Humane Care and Use of Laboratory Animals. The nutritional plan utilized by the ONPRC is based on National Research Council recommendations and supplemented with a variety of fruits, vegetables, and other edible objects as part of the environmental enrichment program established by the Behavioral Services Unit. Animals were socially housed, when possible. All efforts were made to minimize suffering through the use of minimally invasive procedures, anesthetics, analgesics, and environmental enrichment. IceMG and IdeS were administered to macaques intravenously. AAV-Leronlimab vectors were administered intramuscularly, split among 4 injection sites in the quadriceps. Ketamine HCl (3–10 mg/kg IM) with or without dexmedetomidine (5-25 µg/kg IM) was used to sedate macaques for procedures, including IceMG and IdeS IV infusions, AAV-Leronlimab vector IM administration, and blood collection. For euthanasia, macaques were sedated with Ketamine HCl (20 mg/kg) for transportation to the necropsy suite then euthanized with sodium pentobarbital (25 mg/kg IV). Depth of anesthesia was assessed by loss of palpebral, corneal, withdrawal, and gag reflexes, followed by exsanguination via the distal aorta, or right atrium or alternate major vessel such as femoral artery. This euthanasia method is acceptable under the AVMA guidelines for Euthanasia of Animals.

### 2.2 Sample processing

Macaque blood and tissues were collected as previously described (24). Whole blood was collected in EDTA tubes, centrifuged at 1860rcf for 10 minutes to isolate plasma, and blood cells were resuspended in PBS for CCR5 receptor occupancy measurements. Plasma was clarified by centrifugation at 830rcf for 4 minutes, aliquoted, and frozen at -80°C. Lymph nodes and spleen were collected in R10 media (RPMI1640, 10% fetal bovine serum, antibiotic/antimycotic (HyClone), 2mM L-glutamine, 1mM sodium pyruvate), diced and mashed through 70-micron cell strainers to collect single-cell suspensions. Spleen cells were ACK-treated to lyse red blood cells. Lymphocytes in lymph node and spleen preparations were counted using a Horiba ABX Pentra 60 C +.

### 2.3 Plasma immunoglobulin measurements and IgG subtype cleavage assay

Plasma samples were heat-inactivated at 56°C for 1 hour and clarified by centrifugation. For the IgG subtype cleavage assay shown in **Supplementary Figure S1A**, 10µg of reference anti-CD8alpha antibodies representing each rhesus IgG subtype [IgG1 (RRID: AB_2819330), IgG2 (RRID: AB_2819331), IgG3 (RRID: AB_2819332), IgG4 (RRID: AB_2819333)], polycloncal monkey IgG (Rockland, 017-0102), or polyclonal monkey IgM (Rockland, 017-0107) were incubated with the indicated concentration of IceMG or IdeS for 1 hour at 37°C. To measure Ig cleavage, resulting samples were incubated at 95°C for 5 min in 1x LDS loading buffer supplemented with 2.5% β-mercaptoethanol, separated by SDS-PAGE, and transferred to PVDF membrane. Membranes were blocked with 5% milk powder in Tris-buffered saline with 0.1% Tween 20 (TBST) and incubated for 1 h at room temperature with horseradish peroxidase-conjugated primary antibodies for human IgM Fc (1:5, 000; Proteintech, HRP-66484, RRID: AB_2883841) or IgG Fc (1:10, 000; Invitrogen, 31423, RRID: AB_228409). Membranes were then washed 3× with TBST and incubated for 1 min with enhanced chemiluminescence substrate (Thermo Fisher Scientific, 34049). Coomassie-stained gels and immunoblots were visualized on a Bio-Rad ChemiDoc imaging system and quantitated using Bio-Rad Image Lab v.6.1.0. Briefly, densitometry was used to calculate volumes of uncleaved (intact) and cleaved IgG/IgM bands. The percent uncleaved (intact) relative to cleaved was calculated as a percent uncleaved (intact). Additionally, the percent uncleaved (intact) relative to pre-treatment baseline at day 0 was also calculated as percent uncleaved (intact) relative to day 0.

### 2.4 AAV vectors and AAV9 NAb titers

AAV9-Leronlimab vectors were produced as previously described (2, 5, 25–27) HEK-293 cells were transfected with the rAAV vector plasmid encoding the indicated Leronlimab transgene and two helper plasmids. AAV was harvested from cells and supernatant, purified by three sequential CsCl centrifugation steps, quantified by digital droplet PCR, and assessed for purity by silver-stained SDS-PAGE. Two vectors were used in this study (1): AAV9-huLeron encodes parental Leronlimab under a CMV promoter with a P2A heavy/light chain linker and 3’ microRNA target sequences to limit antigen-presenting cell expression (2), and (2) AAV9-macLS-Leron encodes a macaque IgG4 Fc Leronlimab variant containing silencing LALA mutations (L234A/L235A), stabilizing S131C, and LS (M428L/N434S) half-life-extending mutations, under a CMV promoter with a P2A heavy/light chain linker and 3’ microRNA targets. AAV9 NAb 50% inhibitory dilution (ID_50_) titers in plasma samples were determined as previously described (28). Briefly, heat-inactivated plasma samples were tested in a luciferase reporter system where AAV reporter virus was pre-incubated with plasma dilutions and then added to AAV9-susceptible CHO-Lec2 cells (species: *Cricetulus griseus*; ATCC Cat#CRL-1736; batch #70043061) infected with Adeno Helper virus. Luciferase expression was quantified 48 hours later using the Biotek Synergy Mx luminometer. AAV9 NAb ID_50_ for each sample was determined based on global fitting of the data obtained from 2-fold serial sample dilutions run in duplicate. Percent relative to baseline was calculated by dividing the timepoint result by the baseline (day 0) result and multiplying by 100%.

### 2.5 SHIV infection, Env-binding IgG measurements, and plasma viral loads

Macaques were infected with the following CCR5-tropic SHIV virus stocks in previous studies. NHP-2, NHP-4, NHP-5, NHP-6, NHP-8, and NHP-13 received SHIV_SF162P3_ intrarectally (668 TCID_50_ by TZM-bl; stock SHIV162P3–2016 R922 derived Harvest 4, 2.67 x 10^5^ TCID_50_/mL in TZM-bl, 1.28 x 10^3^ TCID_50_/mL in rhesus PBMC, 1 x 10^9^ copies/mL, 173 ng/mL p27, harvested 16 September 2016). NHP-7, NHP-9, and NHP-10 received SHIV_SF162P3_ intrarectally (668 TCID_50_ by TZM-bl; stock SHIV162P3–2016 R922 derived Harvest 2, 2.67 x 10^5^ TCID_50_/mL in TZM-bl, 3.23 x 10^3^ TCID_50_/mL in rhesus PBMC, 1 x 10^9^ copies/mL, 186 ng/mL p27, harvested 09 September 2016). NHP-1, NHP-3, NHP-12, and NHP-14 received SHIV-AD8EOM IV (10, 000 TCID_50_; stock SP2105A_2019, 2 x 10^5^ TCID_50_/mL, harvested 21 May 2019). NHP-11 received SHIV_SF162P3_ IV (100 TCID_50_; stock 170310-D7, 1.2 x 10^9^ copies/mL, 6.7 x 10^5^ TCID_50_/mL, harvested 17 March 2017). SHIV Env-binding IgG measurements were performed as previously described for SIVmac239 Env-binding IgG (29), with the following modifications. Plates were coated with 0.5 µg/mL of HIV SF_162_ Env gp140 trimer (BEI Resources, cat# ARP-12026), and HIV Env-specific VRC07 antibody was used for the standard curve. A minimum of four dilutions of each sample were run in duplicate. Percent relative to baseline was calculated by dividing the timepoint result by the baseline (day 0) result and multiplying by 100%. SHIV plasma viral loads were measured as previously described (30).

### 2.6 Anti-drug antibody, Leronlimab quantification, and CCR5 receptor occupancy assays

Leronlimab-specific ADA titers were measured by ELISA as previously described (5). Plates were coated huLeron, macLS-Leron (IgG4-macLS-Leronlimab), or IgG1-macLS-Leron (IgG1-macLS-Leronlimab) for detection of corresponding ADAs. Notably, ADAs against huLeron and macLS-Leron (IgG4-macLS-Leron) were detected using anti-rhesus IgG1/3-HRP secondary (NHPRR Cat#PR-1234, RRID: AB_2819289), while ADAs against IgG1-macLS-Leron were detected by bridging ELISA using IgG1-macLS-Leronlimab conjugated to HRP as the secondary. Thus, huLeron and macLS-Leron (IgG4-macLS-Leron) ADA assays detect specifically IgG1/IgG3 subclasses of ADA, while the IgG1-macLS-Leronlimab ADA bridging assay detects all potential ADA Ig classes. For the ADA results in **Figures 1K, L**, **2K, L**, and **Supplementary Figure S3A**, the ADA is reported as the raw OD at 450nm after background subtracting of OD at 650nm, and all plasma timepoints shown for each macaque were run in a single assay. For the ADA results in **Figures 1K, L**, **2K, L**, ADA assay corresponding to the Leronlimab that the macaque was previously exposed to: IgG4-macLS-Leron (NHP-1, NHP-2, NHP-3, NHP-4, NHP-5, NHP-6, NHP-8, and NHP-10) or IgG1-macLS-Leron (NHP-9). For the plasma reciprocal dilution ADA titers, the titer is the highest plasma reciprocal dilution where the OD exceeds twice of the OD of a baseline plasma sample taken from prior to Leronlimab exposure and run in the same assay as the test sample (plasma tested in duplicate at 4-fold dilutions from 10 to 10, 240). Plasma and tissue-associated Leronlimab were measured by ELISA as previously described (5, 24), with tissue-associated Leronlimab reported relative to the total protein levels as measured by Pierce BCA Protein assay (Thermo Scientific, cat# 23225). CCR5 receptor occupancy of blood and tissue CD4+ T cells was measured by flow cytometry as previously described (5).

**Figure 1:**
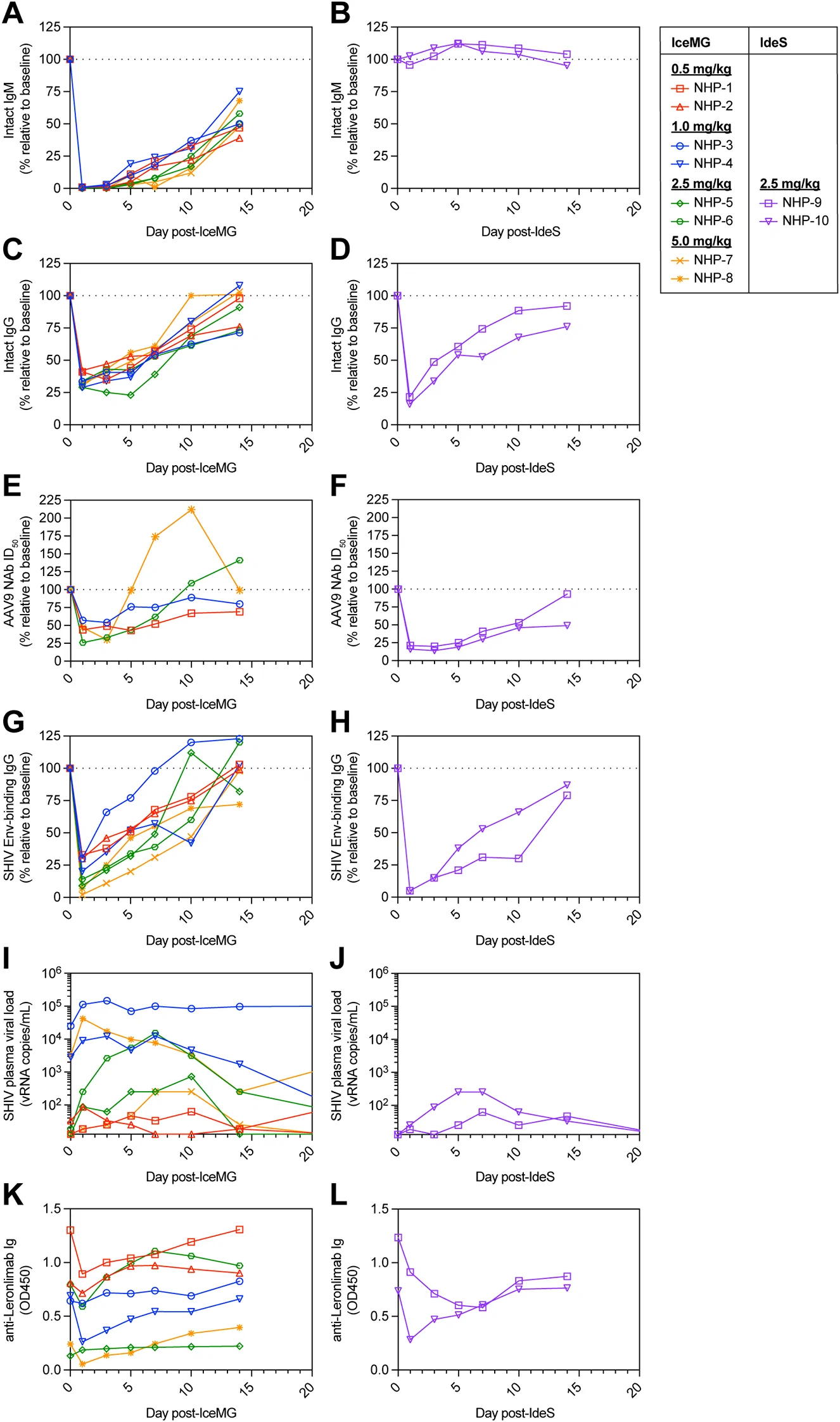
Intravenous IceMG transiently depletes circulating IgM and IgG in rhesus macaques. **(A, B)** Uncleaved (intact) IgM in plasma after IceMG **(A)** or IdeS **(B)**, as percent of baseline. **(C, D)** Uncleaved (intact) IgG in plasma after IceMG **(C)** or IdeS **(D)**, as percent of baseline. **(E, F)** AAV9 NAb ID_50_ titers in plasma after IceMG **(E)** or IdeS **(F)**, as percent of baseline. **(G, H)** SHIV Env-binding IgG concentrations in plasma after IceMG **(G)** or IdeS **(H)**, as percent of baseline. **(I, J)** SHIV plasma viral loads after IceMG **(I)** or IdeS **(J)**. **(K, L)** Leronlimab-specific ADA levels in plasma after IceMG **(K)** or IdeS **(L)**. Horizontal dotted lines **(A, H)** indicate baseline levels (100%).

**Figure 2:**
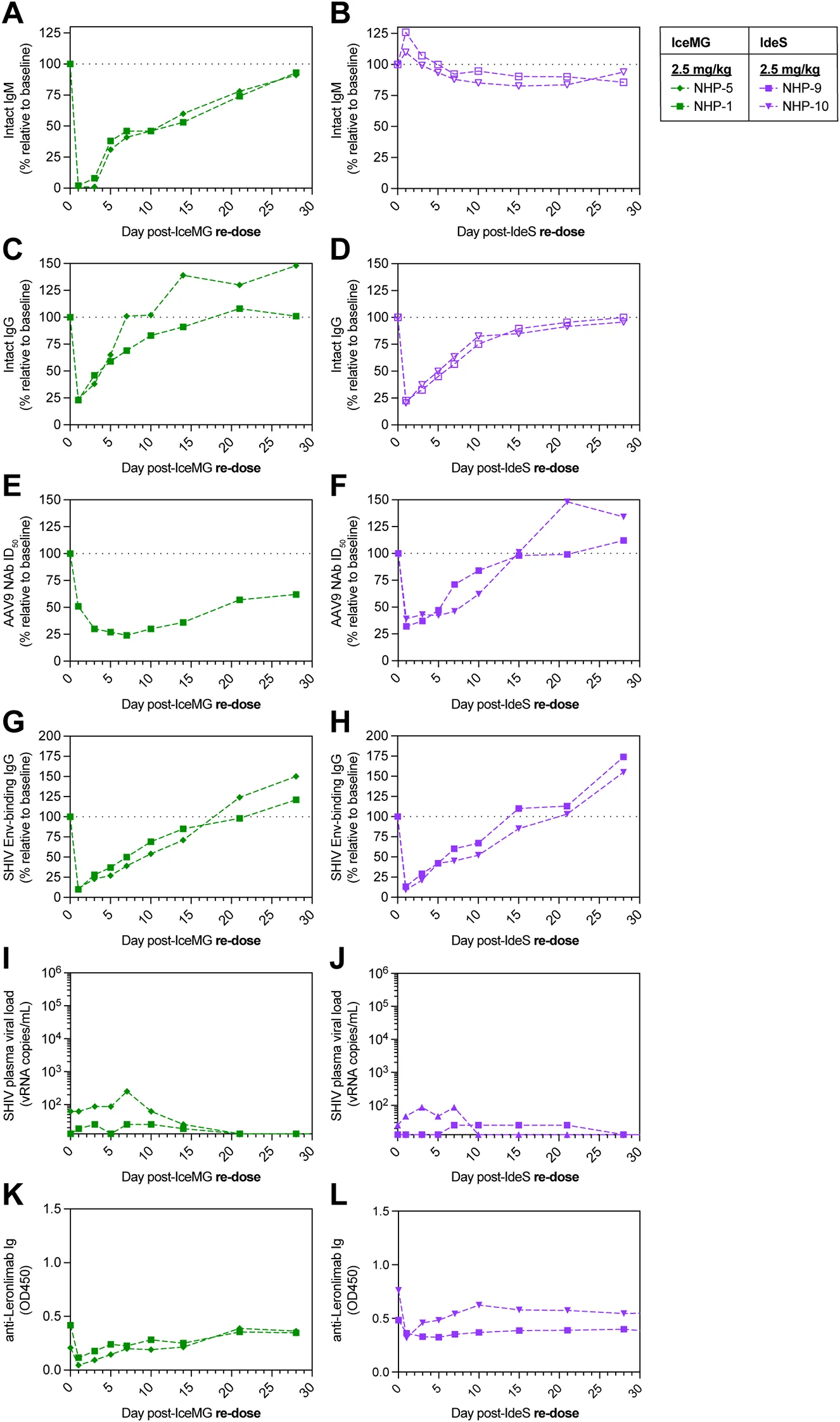
A second administration of IceMG and IdeS is similarly effective to initial dosing. **(A, B)** Uncleaved (intact) IgM in plasma after IceMG **(A)** or IdeS **(B)** re-dose, as percent of baseline. **(C, D)** Uncleaved (intact) IgG in plasma after IceMG **(C)** or IdeS **(D)** re-dose, as percent of baseline. **(E, F)** AAV9 NAb ID_50_ titers in plasma after IceMG **(E)** or IdeS **(F)** re-dose, as percent of baseline. **(G, H)** SHIV Env-binding IgG concentrations in plasma after IceMG **(G)** or IdeS **(H)** re-dose, as percent of baseline. **(I, J)** SHIV plasma viral loads after IceMG **(I)** or IdeS **(J)** re-dose. **(K, L)** Leronlimab-specific ADA levels in plasma after IceMG **(K)** or IdeS **(L)** re-dose. Horizontal dotted lines **(A–H)** indicate baseline levels (100%).

### 2.7 Statistical analyses and graphs

Statistical analyses were performed using GraphPad Prism version 10.6. Sidak’s multiple comparison test was used to compare the depletion of circulating IgM, IgG, and Env-specific binding IgG at day 1 post-dosing among IceMG and IdeS dosing groups. Welch’s t-test was used to compare the depletion of circulating IgM, IgG, and Env-specific IgG at day 1 post-dosing among IceMG and IdeS re-dosed macaques. Graphs were made in GraphPad Prism version 10.6. Data points on graphs are average data from replicates; the number of replicates is described above for each assay.

## 3 Results

### 3.1 IceMG cleaves rhesus macaque IgM and IgG *in vivo*

In a previous study, intravenous administration of IceMG to three rhesus macaques at 0.25, 1.0, and 2.5 mg/kg led to complete clearance of circulating IgM and dose-dependent reduction in circulating IgG, with 1.0 and 2.5 mg/kg dosing resulting in similar, but incomplete IgG depletion (23). To confirm these findings in SHIV-infected rhesus macaques and identify the IceMG dose resulting in maximal IgG depletion, we administered IceMG intravenously to 8 SHIV-infected rhesus macaques at doses of 0.5, 1.0, 2.5, and 5.0 mg/kg (n=2 per group) and monitored effects on plasma immunoglobulins through longitudinal blood sampling. For comparison, we also administered 2.5 mg/kg of IgG-cleaving enzyme IdeS to 2 additional SHIV-infected rhesus macaques. All doses of IceMG resulted in cleavage and complete depletion of circulating IgM by 24 hours post-administration, which gradually rebounded over 14 days (**Figure 1A**), while IdeS had no effect on circulating IgM as expected (**Figure 1B**). Consistent with the previous study, IgG was less susceptible to IceMG cleavage than IgM, with intact IgG decreasing to 29-42% of baseline levels (**Figure 1C**). The higher doses of IceMG (1.0, 2.5, and 5 mg/kg) reduced circulating intact IgG levels by 66-71%, while the lowest dose of IceMG (0.5 mg/kg) reduced circulating intact IgG by 58-59%. IdeS administered at 2.5 mg/kg resulted in a statistically similar reduction of intact IgG (78-84% reduction) as the 2.5 mg/kg dose of IceMG (**Figure 1D**; **Supplementary Table S1**). With the exception of one macaque (NHP-5), maximum IgG depletion occurred at 24 hours post-IceMG/IdeS administration and circulating IgG gradually rebounded over 14 days.

Resistance of human IgG2 to IdeS-mediated cleavage has been previously reported (31), and we hypothesized that rhesus macaque IgG2 may also be resistant to IdeS- and IceMG-mediated cleavage, leading to the incomplete cleavage and depletion of circulating IgG observed in our rhesus macaque studies. To test this, we incubated a representative antibody engineered with rhesus macaque IgG1, IgG2, IgG3, and IgG4 constant regions with IceMG or IdeS *in vitro*. Compared to the other IgG subclasses, rhesus macaque IgG2 was resistant to cleavage by both IceMG and IdeS (**Supplementary Figure S1A**). Thus, incomplete cleavage and depletion of plasma IgG *in vivo* in rhesus macaques likely resulted from resistance of rhesus macaque IgG2 to IceMG and IdeS cleavage.

### 3.2 IceMG and IdeS reduce AAV- and SHIV-specific humoral immunity and increase SHIV viremia

Next, we examined the impact of IceMG and IdeS on virus-specific humoral immunity. A subset of the IceMG- and IdeS- dosed macaques had previously received AAV9 or AAV.cc47, an evolved AAV9 variant (32), gene therapy vectors, and thus had pre-existing AAV9-specific NAbs circulating in plasma prior to enzyme administration. AAV9 neutralization assays of longitudinal plasma samples demonstrated that all doses of IceMG reduced AAV9-specific neutralizing antibody ID_50_ titers by 24 hours post-administration (**Figure 1E**; **Supplementary Figure S1B**), with depletion to 26-57% of baseline ID_50_ titers. Similarly, IdeS reduced AAV9-specific neutralizing antibody (NAb) ID_50_ titers to 16-21% of baseline ID_50_ titers by 24 hours post-administration (**Figure 1F**; **Supplementary Figure S1C**). Notably, all macaques maintained the lower AAV9 NAb titers from 24 hours through 72 hours (day 3) post-administration, after which titers began rebounding.

All IceMG- and Ide-S dosed macaques were chronically SHIV-infected with varying levels of SHIV plasma viremia prior to enzyme administration. Thus, we measured SHIV Env-binding IgG concentrations in longitudinal plasma samples. IceMG decreased plasma concentrations of SHIV Env-binding IgG by 67-98% within 24 hours (**Figure 1G**; **Supplementary Figure S1D**), with the highest doses of IceMG (2.5 and 5.0 mg/kg) leading to >90% reduction. Similarly, 2.5 mg/kg of IdeS reduced SHIV Env-binding IgG plasma concentrations by 95% (**Figure 1H**; **Supplementary Figure S1E**). Consistent with total plasma IgG measurements, SHIV-Env binding IgG concentrations were lowest at 24 hours post-administration and gradually rebounded over 14 days. Notably, depletion of Env-specific IgG was concomitant with increases in SHIV plasma viremia in all IceMG- and IdeS-treated macaques by 24 hours post-administration (**Figures 1I, J**; **Supplementary Figures S1D–E**). In most macaques, SHIV plasma viremia remained elevated for 1 to 2 weeks and then returned to pre-IceMG/IdeS levels, contemporaneous with the rebound of SHIV Env-binding IgG concentrations to baseline levels (**Supplementary Figures S1D–G**). However, two animals exhibited long-term changes in SHIV control after enzyme administration. One macaque (NHP-6) had low levels of plasma viremia prior to IceMG (15–40 SHIV RNA copies/ml), experienced a continuous increase in SHIV plasma viral load peaking at >15, 000 copies/ml on day 7 post-IceMG, and then maintained moderate viremia above baseline for the remaining ~30 weeks of monitoring. Another macaque (NHP-4) had moderate plasma viremia prior to IceMG (2, 000-3, 000 copies/ml), which increased slightly after IceMG but then fell below 500 copies at day 22 post-IceMG and remained below 1, 000 copies/ml for the remainder of the monitoring period.

### 3.3 IceMG and IdeS reduce anti-drug antibody titers

Next, we examined the effects of IceMG and IdeS on pre-existing, circulating anti-drug antibodies (ADAs). A subset of IceMG- and IdeS-dosed macaques had previous exposure to CCR5-specific antibody macLS-Leronlimab (macLS-Leron), either through direct subcutaneous dosing or expression from AAV gene therapy vectors, and had subsequently mounted Leronlimab-directed ADA responses. Thus, we measured circulating anti-Leronlimab ADA levels after IceMG and IdeS administration in longitudinal plasma samples. With the exception of one macaque (NHP-5) that exhibited a very low ADA signal prior to IceMG administration, plasma ADA levels decreased at 24 hours post-IceMG/IdeS administration and then gradually rebounded to baseline levels (**Figures 1K, L**). Thus, ADAs were also susceptible to IceMG and IdeS cleavage.

### 3.4 IceMG and IdeS can be re-administered with similar efficacy

In order to evaluate the ability of IceMG and IdeS to facilitate repeated AAV boosting *in vivo*, we next assessed their efficacy upon re-administration to rhesus macaques. Re-dosing of non-native proteins is often complicated by ADA responses to the initial dose (33, 34), and thus we hypothesized that IceMG and IdeS would not be as effective in a second administration. To test this, we administered a second 2.5 mg/kg intravenous dose of enzyme to four rhesus macaques from the previous study (n=2 per enzyme), waiting 15–44 weeks after the initial enzyme dose. Similar to the initial dosing study, IceMG but not IdeS led to complete cleavage and depletion of IgM from circulation (**Figures 2A, B**). Also consistent with the initial dosing, IceMG and IdeS cleaved and reduced circulating IgG by ~75% in all macaques at 24 hours post-administration, which then gradually rebounded to baseline levels over 1–3 weeks (**Figures 2C, D**). For re-dosed animals with pre-existing AAV9 NAb responses, IceMG and IdeS re-dosing resulted in similar depletion of ID_50_ titers to 30-39% of baseline (**Figures 2E, F**; **Supplementary Figures S2A, B**), which remained low through day 3 and 5 post-administration before gradually rebounding. Re-dosing also reduced SHIV Env-binding IgG plasma concentrations by 87-91% by 24 hours post-administration (**Figures 2G, H**; **Supplementary Figures S2C, D**), and was again accompanied by transient increases in SHIV plasma viremia (**Figures 2I, J**; **Supplementary Figures S2C–F**), although plasma viral load changes were slightly delayed and more modest than observed with the initial IceMG/IdeS dosing. Finally, Leronlimab-specific ADA levels in plasma also temporarily decreased after IceMG and IdeS re-dosing (**Figures 2K, L**). Thus overall, a second administration of IceMG and IdeS showed similar efficacy to the initial dose.

### 3.5 IceMG enables successful AAV transduction in AAV-seropositive macaques

Given the substantial depletion of AAV-specific humoral immunity observed after IceMG administration *in vivo*, we next sought to test the ability of IceMG to facilitate AAV-mediated gene transfer in AAV-seropositive, SHIV-infected macaques that had previously received AAV9 vectors. We hypothesized that IceMG pre-dosing would transiently reduce AAV9 NAbs and thus enable successful transduction by an AAV9 vector boost. First, we performed a pilot experiment in a SHIV-infected Mauritian cynomolgus macaque (NHP-11) possessing a substantial AAV9 NAb plasma titer (ID_50_ > 10^4^), which developed after intramuscular dosing of AAV9 vectors encoding the humanized version of CCR5-specific IgG4 antibody Leronlimab (huLeron) (5). For the current study, we administered a 2.5 mg/kg intravenous dose of IceMG three days prior to an intramuscular dose of AAV9 encoding macLS-Leron and monitored effects longitudinally via blood draws (**Figure 3A**). We selected the 2.5 mg/kg dose of IceMG because maximal depletion of AAV9-specific neutralizing antibodies was observed with 2.5 and 5.0 mg/kg doses in the pilot study (**Figure 1E**). We selected the macLS-Leron transgene for the boosting AAV9 vector because while the CCR5-binding domains of macLS-Leron and huLeron are identical, macLS-Leron protein can be distinguished from huLeron protein in detection assays due to the differences between macaque and human IgG4 constant regions, allowing for discrimination between protein produced by the boosting AAV9 vector (macLS-Leron) and protein produced by the initial AAV9 vector (huLeron). Similar to the rhesus macaque pilot studies, IceMG dosing resulted in complete cleavage and depletion of circulating IgM and substantial cleavage and depletion of circulating IgG on day 3 post-IceMG (**Figures 3B, C**). Critically, IceMG reduced the AAV9 NAb ID_50_ titer in plasma by 90%, from 36,986 pre-IceMG to 3,679 on day 3 post-IceMG (**Figures 3D, E**), and thus AAV9 NAbs were substantially reduced on the day of AAV9-macLS-Leron administration. Notably however, the AAV9 NAb titer increased above 2, 000% of baseline (ID_50_ = 746, 231) within two weeks of AAV9 boost, in contrast to the gradual rebound of AAV9 NAbs observed with IceMG alone in the pilot studies and presumably resulting from a rapid recall response upon re-exposure to AAV9 capsid. In order to assess *in vivo* transduction by the boosting AAV9-macLS-Leron dose, we measured longitudinal plasma concentrations of macLS-Leron and observed emergence of free macLS-Leron protein in circulation at day 33 post-AAV9-macLS-Leron (**Figure 3F**), confirming successful transduction by the AAV9 vector boost after pre-dosing with IceMG.

**Figure 3:**
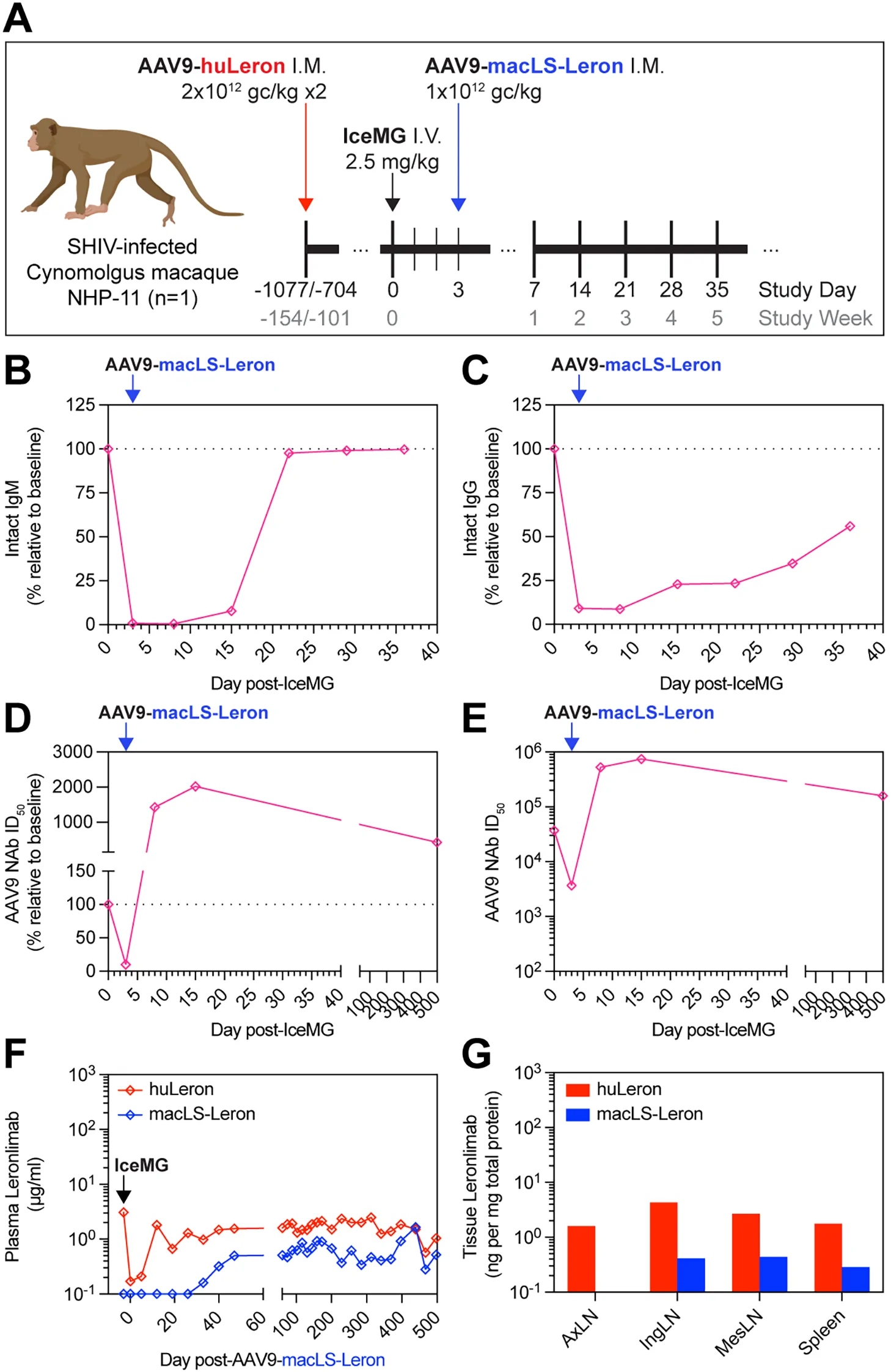
Successful AAV gene transfer in an AAV-seropositive cynomolgus macaques after IceMG pre-dosing. **(A)** Study design. I.M., intramuscular; I.V., intravenous. **(B)** Uncleaved (intact) IgM in plasma, as percent of baseline. **(C)** Uncleaved (intact) IgG in plasma, as percent of baseline. **(D, E)** AAV9 NAb ID_50_ titers in plasma, percent of baseline **(D)** or raw **(E)**. **(F)** Plasma Leronlimab protein concentrations. **(G)** Tissue-associated Leronlimab levels at time of euthanasia (day 500 post-IceMG). Bars not shown indicate Leronlimab was undetectable despite detection of total protein. LN, lymph node. Horizontal dotted lines **(B–D)** indicate baseline levels (100%). Created in BioRender. Wu H. (2026).

Prior to this study, the macaque had detectable plasma huLeron, which had been maintained for ~3 years since the initial AAV9-huLeron dosing and had mediated moderate control of SHIV replication characterized by periodic low-level blips in plasma viremia (5). Since huLeron is a humanized IgG4 antibody, we suspected it would be susceptible to IceMG cleavage, and indeed we observed a transient dip in huLeron plasma concentrations after IceMG, which returned to baseline levels by day 15 post-IceMG (**Figure 3F**). This macaque did not mount a substantial ADA response to huLeron after the initial AAV9-huLeron vector (**Supplementary Figure S3A**), and we did not observe any ADA response to either huLeron or macLS-Leron after IceMG and AAV9-macLS-Leron boost (**Supplementary Figures S3B–D**). Similar to the pilot studies in rhesus macaques, IceMG transiently reduced SHIV Env-binding IgG plasma concentrations by 79% and transiently increased SHIV plasma viremia from undetectable to 2, 000 copies/ml (**Supplementary Figures S3E, F**). Due to Leronlimab’s ability to block CCR5 and control SHIV replication in macaques (5, 24, 30), we hypothesized that successful transduction by AAV9-macLS-Leron and production of macLS-Leron protein would bolster CCR5 blockade and enhance SHIV control once the transient effects of IceMG passed. However, despite maintenance of detectable plasma huLeron and macLS-Leron through day 497 post-AAV9-macLS-Leron, blips of SHIV plasma viremia continued in this macaque (**Supplementary Figure S3G**). At time of euthanasia, free huLeron was low but detectable across lymph nodes and spleen, while macLS-Leron was lower and inconsistently detected (**Figure 3G**), suggesting that continual blips of plasma viremia may have resulted from insufficient AAV-produced Leronlimab in key sites of SHIV replication.

To confirm the utility of IceMG for AAV-mediated gene transfer in AAV-seropositive macaques, we performed a study with four SHIV-infected rhesus macaques that had previously received AAV9-macLS-Leron and thus possessed high plasma titers of AAV9 NAbs (ID_50_ ranging from 10^3^-10^4^). We administered a boosting intramuscular dose of AAV9-huLeron to all four macaques, but only two received 2.5 mg/kg IceMG pre-dosing while the remaining two served as untreated controls (n=2 per group) (**Figure 4A**). IceMG completely depleted circulating IgM and reduced circulating IgG by 69-83% in the two IceMG-treated macaques, while the untreated macaques maintained stable levels of circulating IgM and IgG (**Figures 4B, C**). Plasma AAV9 NAb titers in the IceMG-treated macaques decreased by 42-56% on day 3 post-IceMG, the day of AAV9-huLeron administration, but remained steady in the untreated macaques (**Figures 4D, E**). Similar to the cynomolgus macaque described above, the boosting dose of AAV9 was followed by massive increases in AAV9 NAb titers in all four animals, increasing to 2,000 to 21,000% of baseline levels by day 11 (ID_50_ ranging from 93,545 to 897,628). To assess successful gene transfer by the boosting AAV9-huLeron dose, we measured longitudinal plasma concentrations of free huLeron protein and detected plasma huLeron protein within 2–3 weeks post-AAV boost only in the macaques pre-dosed with IceMG (**Figures 4F, G**). In contrast, huLeron remained undetectable in the macaques that did not receive IceMG (**Figures 4H, I**), suggesting IceMG facilitated successful gene transfer in the treated macaques. However, huLeron protein plasma levels in the IceMG-treated macaques were not sustained and fell below detectable levels at 6 weeks post-boost.

**Figure 4:**
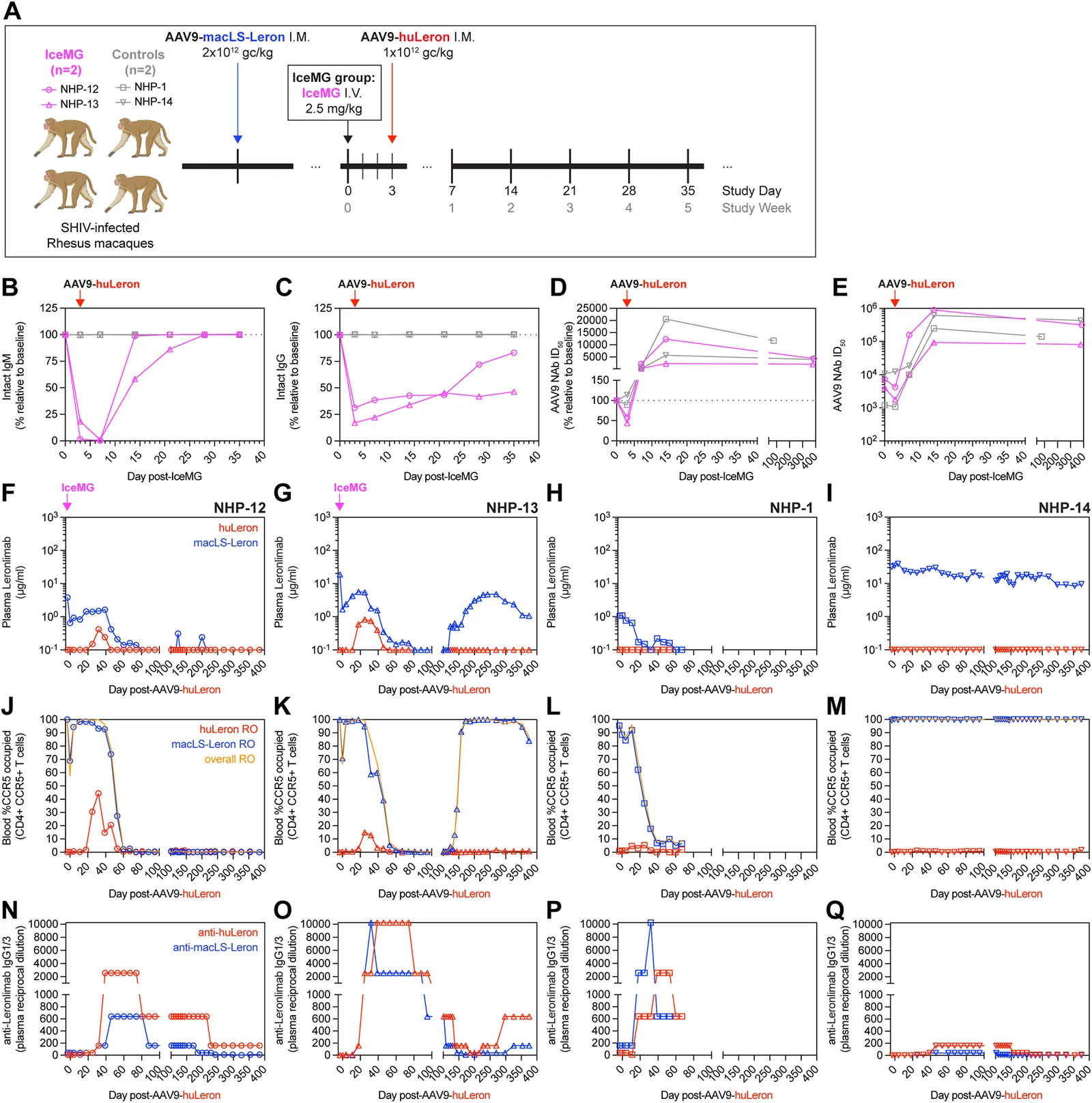
IceMG facilitates successful AAV gene transfer in an AAV-seropositive rhesus macaques. **(A)** Study design. I.M. = intramuscular. I.V. = intravenous. **(B)** Uncleaved (intact) IgM in plasma, as percent of baseline. **(C)** Uncleaved (intact) IgG in plasma, as percent of baseline. **(D, E)** AAV9 NAb ID_50_ titers in plasma, percent of baseline **(D)** or raw **(E)**. **(F–I)** Plasma Leronlimab protein concentrations in IceMG pre-dosed macaques NHP-12 **(F)** and NHP-13 **(G)** and in untreated controls NHP-1 **(H)** and NHP-14 **(I)**. **(J–M)** Leronlimab CCR5 receptor occupancy (RO) of blood CD4+ T cells in IceMG pre-dosed macaques NHP-12 **(J)** and NHP-13 **(K)** and in untreated controls NHP-1 **(L)** and NHP-14 **(M)**. **(N–Q)** Leronlimab-specific ADA plasma reciprocal dilution titers in IceMG pre-dosed macaques NHP-12 **(N)** and NHP-13 **(O)** and in untreated controls NHP-1 **(P)** and NHP-14 **(Q)**. Horizontal dotted lines **(B–E)** indicate baseline levels (100%). Created in BioRender. Wu H. (2026).

Prior to this study, all four macaques had detectable plasma macLS-Leron protein as a result of prior AAV9-macLS-Leron gene therapy. To assess effects of IceMG on macLS-Leron, the rhesus macaque IgG4 antibody circulating in the macaques at the time of dosing, we also measured longitudinal plasma concentrations of free macLS-Leron protein. As expected, macLS-Leron protein was transiently reduced in the macaques that received IceMG, reaching nadir concentrations at day 3 post-IceMG and then gradually rebounding over the following 3–5 weeks (**Figures 4F, G**). However, macLS-Leron plasma concentrations in both IceMG-treated macaques then quickly decreased again, falling below detectable levels by week 12 and remaining undetectable for weeks. As expected, neither untreated control macaque experienced the immediate, transient depletion of circulating macLS-Leron observed in the IceMG-treated macaques (**Figures 4H, I**). However, while one untreated control (NHP-14) maintained steady macLS-Leron plasma levels throughout the study as expected (**Figure 4I**), the other (NHP-1) experienced a delayed but sustained clearance of plasma macLS-Leron similar to that observed in the IceMG-treated macaques (**Figure 4H**).

We also monitored CCR5 receptor occupancy by Leronlimab in longitudinal blood samples because the vast majority of AAV-produced Leronlimab protein would be expected to bind target CCR5 molecules on the surface of circulating cells. Similar to plasma Leronlimab detection, this assay distinguishes between CCR5 occupied by huLeron and macLS-Leron, and also measures overall CCR5 receptor occupancy. Consistent with plasma Leronlimab measurements, huLeron occupancy of CCR5 was transiently detected in the IceMG-treated macaques at timepoints of detectable plasma huLeron, but disappeared after clearance of plasma huLeron (**Figures 4J, K**). Untreated control macaques remained negative for huLeron CCR5 receptor occupancy throughout (**Figures 4L, M**), providing further evidence that IceMG enabled gene transfer after AAV boost in the treated macaques. CCR5 receptor occupancy by macLS-Leron also trended with plasma macLS-Leron protein levels. IceMG-treated macaques experienced a transient dip in macLS-Leron CCR5 RO immediately post-IceMG, which rebounded back to 100% before dropping coincident with clearance of plasma macLS-Leron (**Figures 4J, K**). The control macaque that cleared macLS-Leron protein after AAV boost also cleared macLS-Leron from cell surface CCR5 (NHP-1) (**Figure 4L**). In contrast, the control macaque that maintained steady macLS-Leron plasma levels also maintained 100% CCR5 receptor occupancy by macLS-Leron (NHP-14) (**Figure 4M**).

We hypothesized that the observed clearance of plasma huLeron and macLS-Leron protein in three of the studied macaques after AAV9-huLeron boost was the result of Leronlimab-specific ADAs. Indeed, clearance of plasma Leronlimab protein and blood CD4+ T cell CCR5 receptor occupancy in the three macaques was temporally associated with increasing titers of circulating Leronlimab-specific ADAs, a response that was cross-reactive to both huLeron and macLS-Leron (**Figures 4N–P**). In contrast, the macaque that maintained macLS-Leron in circulation (NHP-14) did not mount significant ADA responses to either huLeron or macLS-Leron (**Figure 4Q**). Of note, two macaques that exhibited marked post-AAV9-huLeron ADA (NHP-12 and NHP-1) had previously mounted macLS-Leron-directed ADA responses after AAV9-macLS-Leron gene therapy, but macLS-Leron ADA titers had waned and remained very low or undetectable for >100 weeks leading up to IceMG and AAV re-dosing (**Supplementary Figure S4A**). We previously observed that ADA-mediated clearance of Leronlimab in AAV9-Leronlimab-dosed macaques can be followed by Leronlimab transgene re-emergence once ADA titers wane (5). Indeed, in one macaque (NHP-13), ADA titers declined and macLS-Leron protein re-emerged in plasma and bound to cell surface CCR5 seven weeks after macLS-Leron clearance, at 18 weeks post-AAV9-huLeron boost (**Figures 4G, K, O**). Despite waning of both huLeron- and macLS-Leron-directed ADA levels, only macLS-Leron protein re-emerged, and we did not observe re-emergence of huLeron protein or CCR5 receptor occupancy for the remainder of the monitoring period. Further, Leronlimab-specific ADAs spontaneously rebounded in this macaque around 32 weeks post-AAV9-macLS-Leron, which was followed by decreasing macLS-Leron protein concentrations and decreasing CCR5 receptor occupancy.

Finally, we measured changes in SIV-specific humoral immunity and SHIV control. Prior to the study, all four macaques were stringently controlling SHIV plasma viremia with undetectable plasma viral loads and complete (100%) or near complete (95%) CCR5 receptor occupancy of blood CD4+ T cells by macLS-Leronlimab. As expected, SHIV Env-binding IgG plasma concentrations transiently decreased by 81-88% and SHIV plasma viral loads transiently increased in the IceMG-treated macaques, while the untreated macaques maintained steady plasma levels of SHIV Env-binding IgG and undetectable SHIV plasma viral loads (**Supplementary Figures S4B-D**). Three macaques, both IceMG-treated macaques and one untreated control, were monitored for over a year after AAV9-huLeron boost. While the effects of IceMG were transient, ADA-mediated clearance of plasma Leronlimab from one IceMG-treated macaque (NHP-12) was eventually followed by blips of plasma viremia, indicating some loss of control over SHIV replication. In contrast, the IceMG-treated macaque experiencing macLS-Leron transgene re-emergence (NHP-13) and the untreated control macaque that maintained circulating levels of macLS-Leron throughout (NHP-14) also maintained control of SHIV replication, with the exception of a single blip of plasma viremia in NHP-14.

At time of euthanasia, ~1 year post-AAV boost, we collected blood, lymph nodes, and spleen and measured Leronlimab in tissue supernatants and CCR5 receptor occupancy (**Supplementary Figures S4E–J**). In line with blood measurements, no huLeron protein was detected in tissue supernatants or by receptor occupancy assays in any of the macaques. Also consistent with blood measurements, only control macaque NHP-14 possessed detectable tissue-associated macLS-Leron and complete (100%) CCR5 receptor occupancy of tissue CD4+ T cells, which were coated exclusively by macLS-Leron (**Supplementary Figures S4G, J**). The IceMG-treated macaque with macLS-Leron transgene re-emergence (NHP-13) had lower levels of tissue-associated macLS-Leron and exhibited only partial CCR5 receptor occupancy of tissue CD4+ T cells by macLS-Leron (**Supplementary Figures S4F, I**), consistent with ADA rebound and incomplete CCR5 receptor occupancy of blood CD4+ T cells leading up to euthanasia. The remaining IceMG-treated macaque (NHP-12) did not possess any detectable macLS-Leron in tissue supernatants nor on the surface of tissue CD4+ T cells (**Supplementary Figures S4E, H**). Overall, this study suggests IceMG can enable successful AAV vector gene transfer in AAV-seropositive macaques, but AAV vector re-administration also risks transgene loss due to transgene-directed ADAs.

## 4 Discussion

In this study, we sought to test the ability of IgG/IgM-cleaving enzyme IceMG to overcome AAV humoral immunity and enable AAV gene therapy in AAV-seropositive, SHIV-infected rhesus and cynomolgus macaques. Our results demonstrate that intravenous administration of IceMG cleaves and transiently depletes circulating IgM and IgG *in vivo*. Similar to our previous study in SHIV-naïve cynomolgus macaques (23), cleavage of circulating IgM was complete, while cleavage of circulating IgG was substantial but incomplete. Maximal IgG cleavage by IceMG was achieved with doses at or above 1.0 mg/kg, temporarily decreasing intact circulating IgG levels by 66 to 91%. We also directly compared IceMG to EU-approved IgG-cleaving enzyme IdeS, or imlifidase. Notably, as IceMG is a fusion of IdeZ and IceM, one molecule IceMG (70 kDa) is approximately twice the size of IdeS (35 kDa). Thus, a 2.5 mg/kg dose of IceMG contains approximately half the number of enzyme molecules compared to a 2.5 mg/kg dose of IdeS. Despite this, we observed similar depletion of circulating IgG by the two enzymes when administered at 2.5 mg/kg. Reduction of circulating immunoglobulins by IceMG and IdeS was confirmed by multiple plasma readouts, including AAV9 NAb titers, SHIV Env-binding IgG concentrations, anti-drug antibody titers, and Leronlimab antibody concentrations.

Despite previous reports of anti-drug antibodies targeting IdeS in dosed humans and cynomolgus macaques (21, 35), IdeS maintained efficient cleavage of human plasma IgG in the presence of anti-IdeS antibodies *in vitro* (21), and recent studies found similar efficacy with repeated IdeS and IceMG dosing in rhesus macaques (35, 36). Similarly, we did not observe any reduction in efficacy when IceMG and IdeS were re-administered to rhesus macaques months after the initial dose. We hypothesize that this may be due to susceptilibity of IceMG- and IdeS-specific ADAs to IceMG- and IdeS-mediated cleavage, but this requires further investigation. Overall, preservation of function upon re-dosing suggests IceMG and IdeS could be administered multiple times in an individual’s lifetime and retain efficacy.

The incomplete cleavage and depletion of circulating macaque IgG observed in this study and others (21–23, 35, 37) suggests some forms of IgG are resistant to IceMG, IdeS, and IdeZ enzymatic cleavage. Here, we demonstrate that among rhesus macaque IgG subtypes, IgG2 is resistant to cleavage by both IceMG and IdeS *in vitro*, offering a potential explanation for the persistence of some intact IgG *in vivo* despite increasing IceMG dosages. These results are corroborated by a recent study testing IdeS against rhesus macaque IgG subtypes *in vitro* (38). The authors that originally characterized IdeS also reported that cleavage of human IgG2 was less efficient compared to other human IgG subtypes *in vitro* (31). However, subsequent studies have reported complete cleavage of circulating human IgG by IdeS *in vitro* and *in vivo* (21, 33, 37, 39), while IdeS cleavage was slightly less effective against cynomolgus macaque IgG (21, 37). Thus, IceMG and IdeS may be more effective at cleaving IgG and reducing AAV humoral immunity in humans than the macaques studied here.

In the chronically SHIV-infected rhesus and cynomolgus macaques studied here, IceMG and IdeS reduced SHIV-specific humoral immunity through transient depletion of circulating IgG antibodies targeting Env, the viral envelope protein. This was universally and temporally associated with transient increases in SHIV plasma viral load, regardless of the level of pre-existing viremia, underscoring the role of Env-binding antibodies in containment of SHIV replication. Similar depletion of SIV-specific immunity was recently observed in IdeS-treated pigtail macaques (38), but the authors did not report SIV plasma viral loads. While we did not explore SHIV neutralizing antibody responses in this study, further investigation of the impact of IgG-cleaving enzymes on the functionality of the Env-specific humoral response is warranted, particularly as recent studies have highlighted the role of autologous neutralizing antibodies in post-treatment HIV control (40, 41). Future studies of IceMG and IdeS measuring enzyme-induced changes in plasma viral loads and viral reservoirs in SHIV-infected, cART-treated macaques would be highly informative to the safety and clinical translation of IgG-cleaving enzymes to people living with HIV (PLWH). In addition, IgG-cleaving enzymes like IceMG, IdeZ, and IdeS may be useful tools to interrogate HIV functional cure in experimental macaque models, similar to depletion of CD8+ T cells.

In the SHIV-infected macaques studied here, the depletion of antiviral antibodies targeting AAV and SHIV by IceMG and IdeS was transient, and none of the macaques exhibited signs of immunosuppression prior to or during these studies. However, given the transient increases in SHIV plasma viremia, the safety of immunoglobulin-cleaving enzymes in the setting HIV and other infections should be carefully considered, particularly in immunosuppressed individuals.

Our results demonstrate that IV IceMG pre-dosing reduces AAV9 humoral immunity and enables a second round of successful IM AAV9 vector transduction in rhesus macaques, which is in line with previous studies demonstrating that other IgG-cleaving enzymes IdeS and IdeZ can rescue AAV-mediated gene transfer in AAV-seropositive mice and macaques (21, 22). While the success of AAV gene therapy in AAV-seropositive individuals varies based on the experimental model, pre-existing AAV NAb levels, AAV serotype, AAV route, and AAV dose, intramuscular AAV dosing is typically more successful than intravenous dosing (9–13). Here, in macaques with high titers of AAV9 NAb in plasma, an intramuscular dose of AAV9 gene therapy failed to produce detectable transgene protein unless macaques were pre-dosed with IceMG, demonstrating that IceMG can reduce AAV-specific humoral immunity enough to facilitate successful AAV transduction and gene transfer. This study supports further investigation of IgG-cleaving enzymes for AAV gene therapy in AAV-seropositive individuals, and future studies testing IceMG with other AAV serotypes and intravenous AAV gene therapy are warranted.

In addition, the ability to re-administer AAV vectors is an important consideration for AAV gene therapy because AAV genomes are primarily maintained as episomes, which may ultimately result in waning transgene expression over time as transduced cells divide or die (42, 43). Indeed, we’ve found very gradual waning of Leronlimab protein levels over years in some macaques that received AAV-Leronlimab gene therapy (5). However, AAV vector re-dosing is challenging as the initial AAV vector administration elicits a strong AAV-specific NAb response that can prevent *in vivo* transduction by a subsequent dose of AAV (5, 11, 12, 17–20). Here, we demonstrate that IceMG can facilitate successful IM AAV9 vector re-dosing by lowering circulating AAV9 NAb titers in cynomolgus and rhesus macaques. However, our results also highlight important challenges for re-administration of AAV gene therapy. First, the plasma AAV9 NAb titer surged after the second AAV9 vector administration, in one case by >20, 000% to an ID_50_ titer of nearly 10^6^. While such titers would possibly decline over years, titers remained high at necropsy approximately one year after AAV vector boosting (ID_50_ ranging from 81, 667 to 430, 320). Thus, it is unclear if a second dose of IceMG would lower AAV NAb titers sufficiently to facilitate a third round of AAV vector transduction, although this could perhaps be achieved by multiple days of IceMG dosing leading up to AAV dosing. Second, in some cases, boosting with AAV vectors encoding Leronlimab led to robust Leronlimab-directed ADA responses and subsequent clearance of pre-existing Leronlimab protein from circulation. Thus, for AAV vectors encoding such non-native proteins, AAV vector re-administration risks transgene loss due to the development or recurrence of ADA responses.

Substantial ADA titer increases occurred in three of five macaques after AAV vector boost, including one control macaque that did not receive IceMG pre-dosing (NHP-1). While this macaque had a pre-existing ADA response from the initial dose of AAV9-macLeron that had waned to low circulating titers prior to this study (**Supplementary Figure S4A**) (5), the robust increase in Leronlimab-directed ADAs following AAV vector boosting suggests some low level of transduction and huLeron production by the boosting AAV9-huLeron dose, which potentially reactivated macLS-Leron-directed ADA responses that were cross-reactive to huLeron. It is unclear if huLeron was undetectable in this macaque due to low levels below the assay limit of detection or due to masking by pre-existing bound ADAs. Critically however, this macaque had the lowest pre-study AAV9 NAb titer (ID_50_ = 1, 209), which was lower than the post-IceMG titers in the two IceMG-treated macaques (ID_50_ = 4, 229 and 1, 778) where successful huLeron gene transfer was observed. In contrast, the control macaque with no ADA and no huLeron detection (NHP-14) had a plasma AAV9 NAb titer of 12, 327 ID_50_ on the day of AAV vector boost. Thus, it is plausible that AAV humoral immunity in control macaque NHP-1 was low enough to allow for successful transduction by the boosting AAV vector, without IceMG treatment. Together, these data suggest that circulating AAV9 NAb titers must be below ~5, 000-10, 000 to achieve successful gene transfer with IM AAV vector doses of 1x10^12^ genome copies/kg.

Leronlimab-directed ADA responses after AAV9 re-dose was associated with pre-existing Leronlimab-directed immune responses in some but not all macaques. Two macaques (NHP-12, NHP-1) mounted macLS-Leron-directed ADA responses after initial AAV9-macLS-Leron dosing, which eventually spontaneously resolved and remained near undetectable levels for >100 weeks until AAV9-huLeron re-dosing. Rebounding ADA in these cases likely represents recall of the initial macLS-Leron-directed ADA response due to the significant overlap in protein sequence between huLeron and macLS-Leron, however new huLeron-specific ADAs targeting the human constant regions may also have developed. In contrast, two macaques (NHP-11, NHP-14) did not mount significant Leronlimab-directed ADA responses after initial AAV9-Leronlimab vector dosing or after AAV9-Leronlimab re-dosing. Finally, one macaque (NHP-13) remained completely negative for macLS-Leron-directed plasma ADA after the initial AAV9-macLS-Leron dosing, but subsequently mounted strong Leronlimab-directed ADA responses after AAV-huLeron re-dosing, a response that was cross-reactive to macLS-Leron.

This study has a number of limitations, including the small number of macaques per group and exclusive use of AAV9-Leronlimab vectors administered intramuscularly. Additional studies using other AAV vector capsids, transgenes, and administration routes should be performed to confirm these results. In addition, the dose and IM route used for AAV9 vector boosting here are not expected to activate complement *in vivo* (14–16), and thus we did not measure complement activation *ex vivo* in this study. However, we previously demonstrated that complement activation by AAV9 *in vitro* was reduced in serum samples from AAV9-seropositive, IceMG-treated cynomolgus macaques compared to baseline samples (23). Thus, while the protective efficacy of IceMG pre-dosing against complement-associated toxicities requires further investigation *in vivo*, IceMG offers potential advantages over other IgG-cleaving enzymes and may improve AAV tolerability and efficacy in AAV-seropositive individuals.

## Data Availability

The original contributions presented in the study are included in the article/**Supplementary Material**. Further inquiries can be directed to the corresponding author.
