# Supplementary material for: IgM-and IgG-cleaving enzyme facilitates AAV gene transfer in AAV-seropositive macaques
Source: Front Immunol. 2026 Oct 20;17:1966044. doi: 10.3389/fimmu.2026.1966044 (PMC13727741; doi:10.3389/fimmu.2026.1966044)
Supplement: Supplementary file 1 [file DataSheet1.pdf]

Supplementary Material:

IgM- and IgG-cleaving enzyme (IceMG) facilitates AAV gene transfer in AAV-seropositive macaques

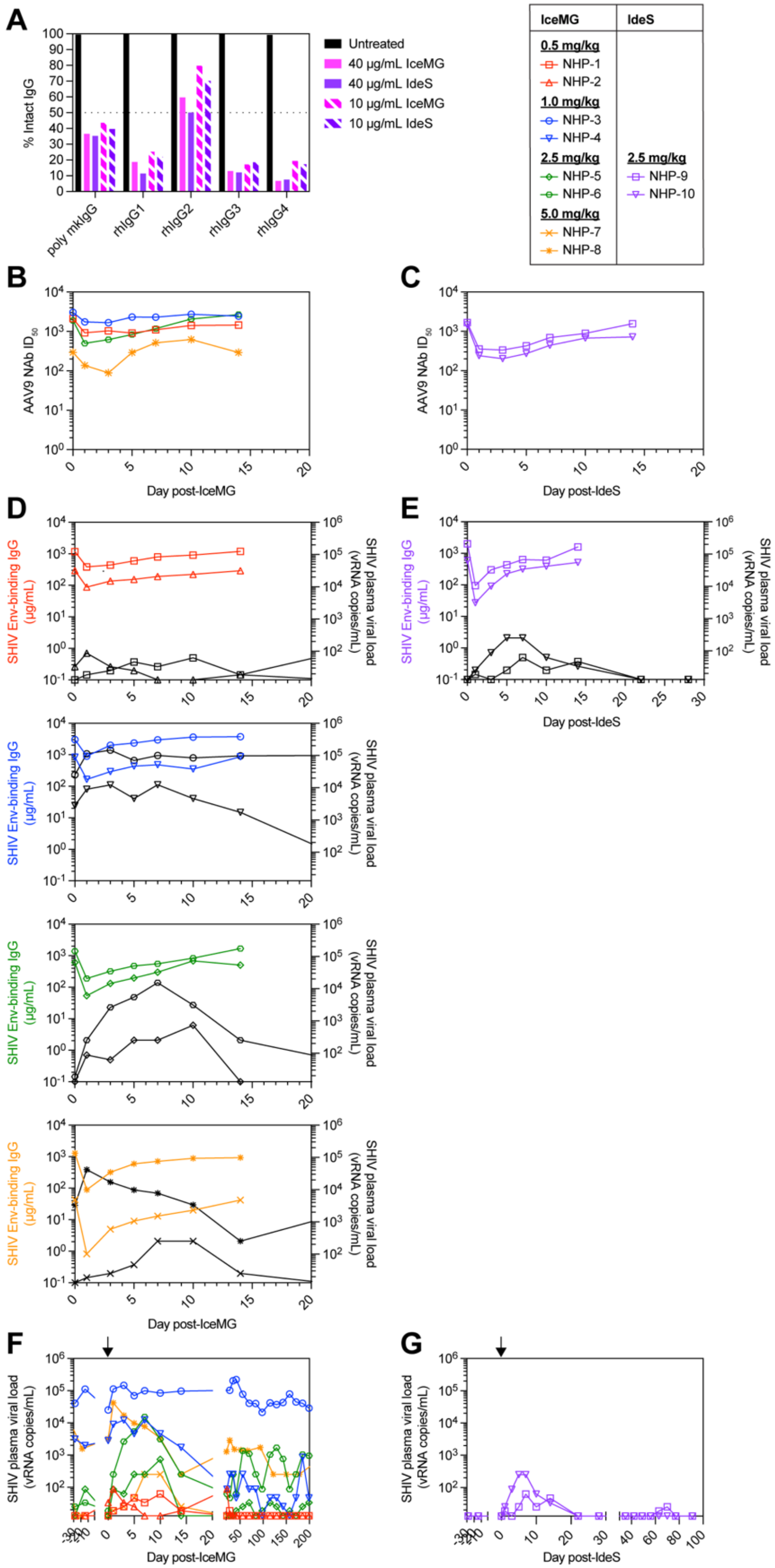
A
% Intact IgG
Untreated
40 µg/mL IceMG
40 µg/mL IdeS
10 µg/mL IceMG
10 µg/mL IdeS
poly mkIgG
rhIgG1
rhIgG2
rhIgG3
rhIgG4
IceMG
IdeS
0.5 mg/kg
NHP-1
NHP-2
1.0 mg/kg
NHP-3
NHP-4
2.5 mg/kg
NHP-5
NHP-6
5.0 mg/kg
NHP-7
NHP-8
2.5 mg/kg
NHP-9
NHP-10
B
C
AAV9 NAb ID50
Day post-IceMG
Day post-IdeS
D
E
SHIV Env-binding IgG (µg/mL)
SHIV plasma viral load (vRNA copies/mL)
Day post-IceMG
Day post-IdeS
F
G
SHIV plasma viral load (vRNA copies/mL)
Day post-IceMG
Day post-IdeS

Figure S1. Additional data related to Figure 1. (A) Percentage uncleaved (intact) IgG after incubation of purified IgG with IceMG and IdeS *in vitro*. **(B-C)** AAV9 NAb ID50 titers of plasma after IceMG (B) or IdeS (C). **(D-E)** Plasma SHIV-binding IgG concentrations (left y-axis, color) versus SHIV plasma viral loads (right y-axis, black) after IceMG (D) or IdeS (E). **(F-G)** Long-term SHIV plasma viral loads after IceMG (F) or IdeS (G). Black arrow indicates timepoint of IceMG or IdeS treatment. Undetectable viral loads are graphed at the x-axis (assay limit of quantification, 13 copies/mL).

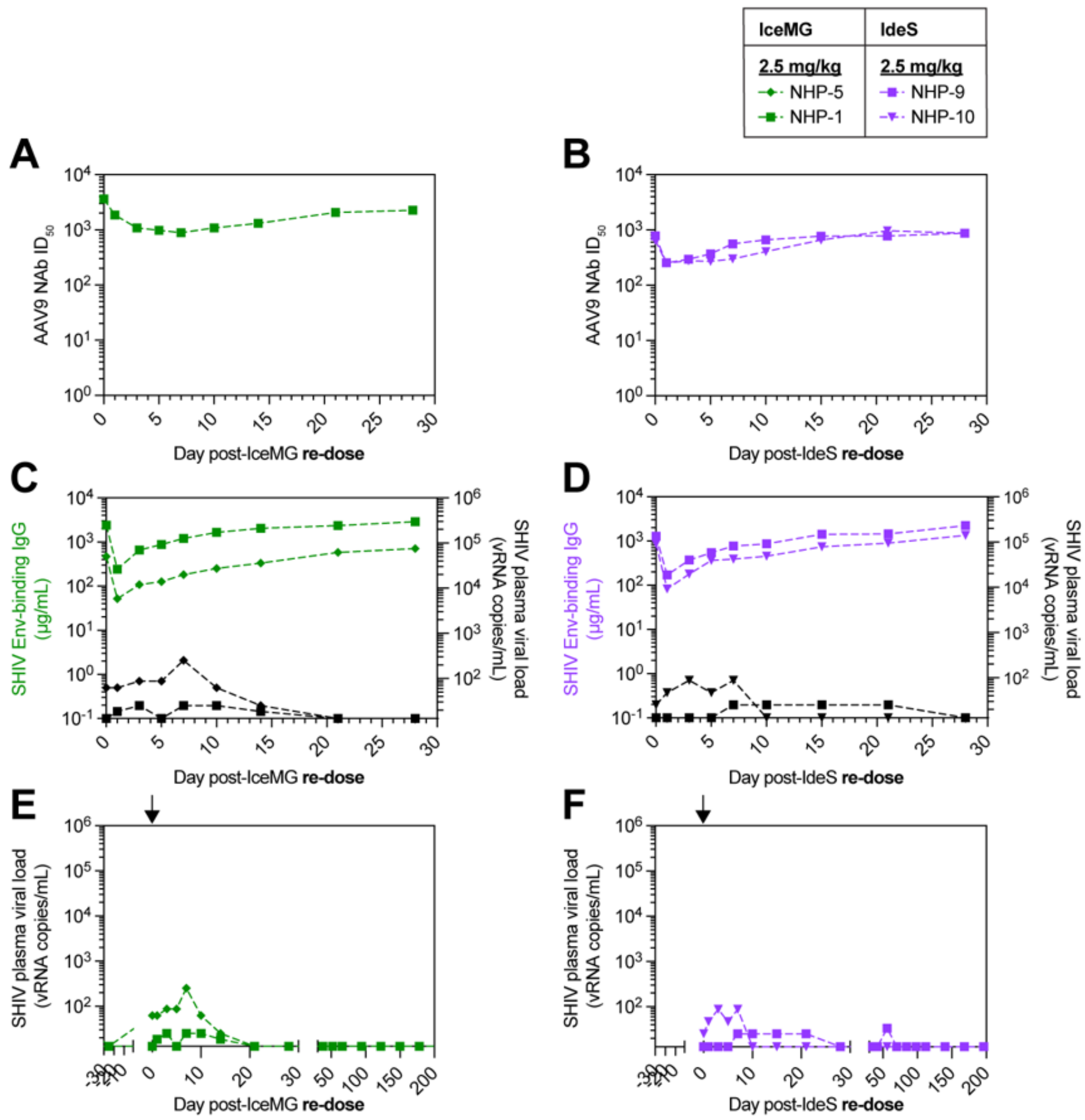

Figure S2. Additional data related to Figure 2. (A-B) AAV9 NAb ID50 titers of plasma after IceMG (A) or IdeS (B) re-dose. **(C-D)** Plasma SHIV-binding IgG concentrations (left y-axis, color) versus SHIV plasma viral loads (right y-axis, black) after IceMG (C) or IdeS (D) re-dose. **(E-F)** Long-term SHIV plasma viral loads after IceMG (E) or IdeS (F) re-dose. Black arrow indicates timepoint of IceMG or IdeS treatment. Undetectable viral loads are graphed at the x-axis (assay limit of quantification, 13 copies/mL).

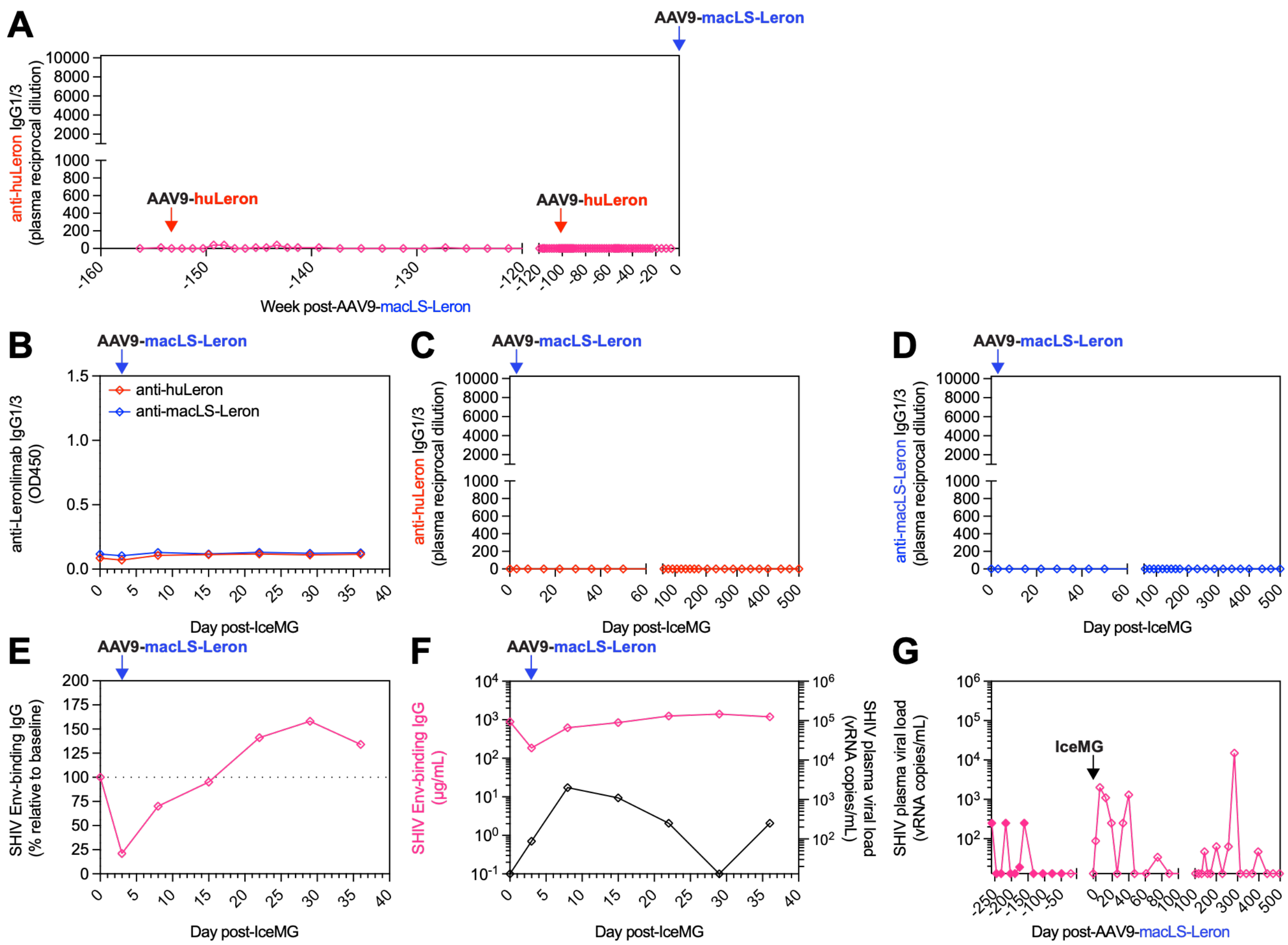

Figure S3. Additional data related to Figure 3. (A) Historical huLeron-specific ADA plasma reciprocal dilution titers. Data originally reported in Wu *et al.* (5). **(B)** Leronlimab-specific ADA levels in plasma. **(C-D)** Leronlimab-specific ADA plasma reciprocal dilution titers. **(E)** SHIV Env-binding IgG concentrations in plasma, as percent of baseline. Horizontal dotted line indicates baseline levels (100%). **(F)** Plasma SHIV-binding IgG concentrations (left y-axis, color) versus SHIV plasma viral loads (right y-axis, black). **(G)** Long-term SHIV plasma viral loads. Undetectable viral loads are graphed at the x-axis (assay limit of quantification, 13 copies/mL). Previously published historical plasma viral loads from Wu *et al.* (5) shown in solid symbols.

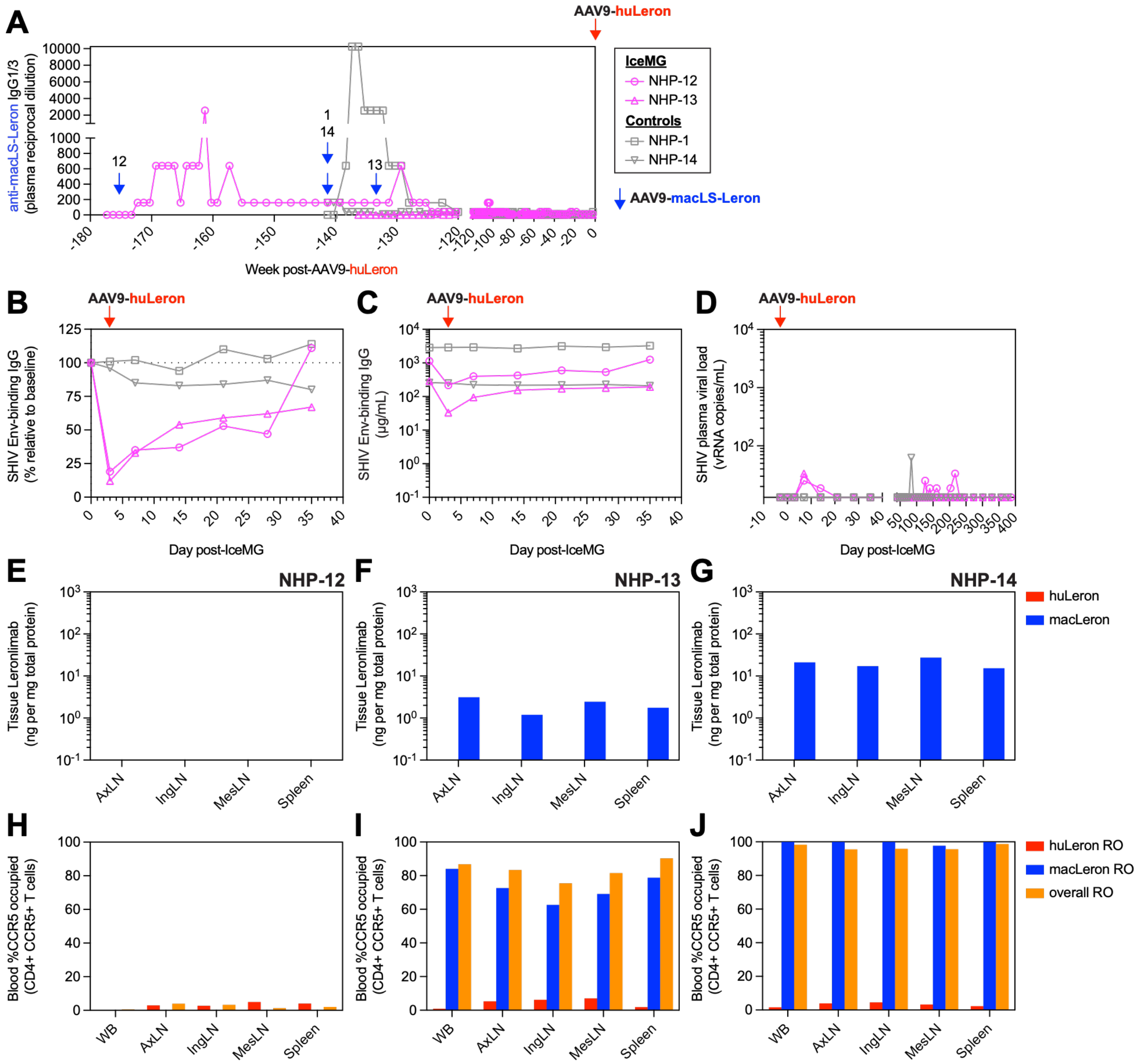

Figure S4. Additional data related to Figure 4. (A) Historical macLS-Leron-specific ADA plasma reciprocal dilution titers. Data originally reported in Wu *et al.* (5). **(B-C)** SHIV Env-binding IgG concentrations in plasma, as percent of baseline (A) or raw (B). Horizontal dotted line indicates baseline levels (100%). **(D)** Long-term SHIV plasma viral loads. Undetectable viral loads are graphed at the x-axis (assay limit of quantification, 13 copies/mL). **(E-G)** Tissue-associated Leronlimab levels at time of euthanasia for NHP-12 (D), NHP-13 (E), and NHP-14 (F) (day 375-384 post-AAV9-huLeron). Bars not shown indicate Leronlimab was undetectable despite detection of total protein. **(H-J)** Leronlimab CCR5 receptor occupancy (RO) of blood and tissue CD4+ T cells at time of euthanasia for NHP-12 (G), NHP-13 (H), and NHP-14 (I) (day 375-384 post-AAV9-huLeron). Bars not shown indicate undetectable receptor occupancy. LN = lymph node.

Table S1. Statistical analyses

**Intact IgM at day 1 (% relative to baseline)**				
	**Dose comparison (mg/kg)**	**Significant**	**P-value**	**Test**
**IceMG vs. IceMG**	0.5 vs. 1	ns	>0.9999	Sidak's multiple comparison
	0.5 vs. 2.5	ns	>0.9999	
	0.5 vs. 5	ns	>0.9999	
	1 vs. 2.5	ns	>0.9999	
	1 vs. 5	ns	>0.9999	
	2.5 vs. 5	ns	>0.9999	
**IceMG vs. IdeS**	0.5 vs. 2.5	****	<0.0001	
	1 vs. 2.5	****	<0.0001	
	2.5 vs. 2.5	****	<0.0001	
	5 vs. 2.5	****	<0.0001	
**IceMG redose vs. IdeS redose**	2.5 vs. 2.5	*	0.0427	Unpaired t-test with Welch's correction
**Intact IgG at day 1 (% relative to baseline)**				
	**Dose comparison (mg/kg)**	**Significant**	**P-value**	**Test**
**IceMG vs. IceMG**	0.5 vs. 1	ns	0.1365	Sidak's multiple comparison
	0.5 vs. 2.5	ns	0.1453	
	0.5 vs. 5	ns	0.1269	
	1 vs. 2.5	ns	>0.9999	
	1 vs. 5	ns	>0.9999	
	2.5 vs. 5	ns	>0.9999	
**IceMG vs. IdeS**	0.5 vs. 2.5	**	0.0047	
	1 vs. 2.5	ns	0.0662	
	2.5 vs. 2.5	ns	0.0625	
	5 vs. 2.5	ns	0.0708	
**IceMG redose vs. IdeS redose**	2.5 vs. 2.5	ns	0.3068	Unpaired t-test with Welch's correction
**SHIV Env-binding IgG at day 1 (% relative to baseline)**				
	**Dose comparison (mg/kg)**	**Significant**	**P-value**	**Test**
**IceMG vs. IceMG**	0.5 vs. 1	ns	0.8333	Sidak's multiple comparison
	0.5 vs. 2.5	*	0.0398	
	0.5 vs. 5	*	0.0106	
	1 vs. 2.5	ns	0.1792	
	1 vs. 5	*	0.0358	
	2.5 vs. 5	ns	0.7775	
**IceMG vs. IdeS**	0.5 vs. 2.5	*	0.0116	
	1 vs. 2.5	*	0.0398	
	2.5 vs. 2.5	ns	0.8333	
	5 vs. 2.5	ns	>0.9999	
**IceMG redose vs. IdeS redose**	2.5 vs. 2.5	ns	0.845	Unpaired t-test with Welch's correction

ns=not significant
